# The Formation of Endoderm-Derived Taste Sensory Organs Requires a *Pax9*-Dependent Expansion of Embryonic Taste Bud Progenitor Cells

**DOI:** 10.1371/journal.pgen.1004709

**Published:** 2014-10-09

**Authors:** Ralf Kist, Michelle Watson, Moira Crosier, Max Robinson, Jennifer Fuchs, Julia Reichelt, Heiko Peters

**Affiliations:** 1 Institute of Genetic Medicine, Newcastle University, International Centre for Life, Newcastle upon Tyne, United Kingdom; 2 Centre for Oral Health Research, School of Dental Sciences, Newcastle University, Newcastle upon Tyne, United Kingdom; 3 Department of Craniofacial Development and Stem Cell Biology, King's College London, Guy's Hospital, London, United Kingdom; 4 Institute of Cellular Medicine, Dermatological Sciences, Newcastle University, Newcastle upon Tyne, United Kingdom; New York University, United States of America

## Abstract

In mammals, taste buds develop in different regions of the oral cavity. Small epithelial protrusions form fungiform papillae on the ectoderm-derived dorsum of the tongue and contain one or few taste buds, while taste buds in the soft palate develop without distinct papilla structures. In contrast, the endoderm-derived circumvallate and foliate papillae located at the back of the tongue contain a large number of taste buds. These taste buds cluster in deep epithelial trenches, which are generated by intercalating a period of epithelial growth between initial placode formation and conversion of epithelial cells into sensory cells. How epithelial trench formation is genetically regulated during development is largely unknown. Here we show that *Pax9* acts upstream of *Pax1* and *Sox9* in the expanding taste progenitor field of the mouse circumvallate papilla. While a reduced number of taste buds develop in a growth-retarded circumvallate papilla of *Pax1* mutant mice, its development arrests completely in *Pax9*-deficient mice. In addition, the *Pax9* mutant circumvallate papilla trenches lack expression of K8 and *Prox1* in the taste bud progenitor cells, and gradually differentiate into an epidermal-like epithelium. We also demonstrate that taste placodes of the soft palate develop through a *Pax9*-dependent induction. Unexpectedly, *Pax9* is dispensable for patterning, morphogenesis and maintenance of taste buds that develop in ectoderm-derived fungiform papillae. Collectively, our data reveal an endoderm-specific developmental program for the formation of taste buds and their associated papilla structures. In this pathway, *Pax9* is essential to generate a pool of taste bud progenitors and to maintain their competence towards prosensory cell fate induction.

## Author Summary

Gustatory perception is an evolutionary ancient sense, and the ability to discriminate toxic and digestible substances is vitally important for all organisms. In mammals, taste perception occurs in taste buds, groups of sensory cells that are housed in various types of taste papillae in the oral cavity. Little is known about the genetic and developmental programs that underlie the different architectures of these papillae. Using mouse models, we identified the transcription factor Pax9 as a major determinant for the development of endoderm-derived taste papillae, which develop in different locations in the back of the oral cavity. In these papillae, Pax9 regulates the expansion of the taste progenitor field, maintains the competence of these progenitors to interact with afferent nerve fibers of the glossopharyngeal nerve, and prevents their differentiation towards epidermal-like epithelial cells. In contrast, Pax9 is not required for the development of ectoderm-derived taste papillae that are distributed over the dorsum of the tongue. Our data reveal that mammals have evolved a specific developmental program to generate taste buds and associated papilla structures in different parts of the oral cavity.

## Introduction

**Figure 1:**
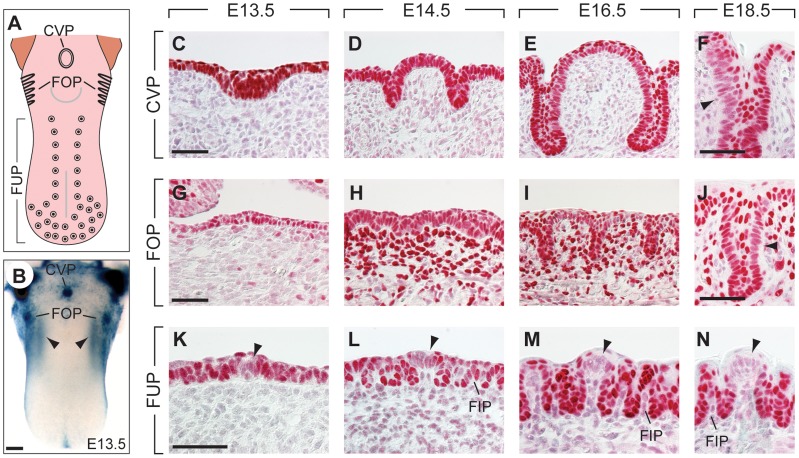
Expression patterns of Pax9 in different taste papillae of the embryonic mouse tongue. (**A**) Drawing showing the localization of the circumvallate papilla (CVP), foliate papillae (FOP), and fungiform papillae (FUP) in the mouse tongue. (**B**) Whole mount X-Gal staining of a *Pax9^+/LacZ^* mouse tongue at embryonic day 13.5 (E13.5). Note that expression is also seen in the mesenchyme adjacent to the developing FOP (arrowheads) and that the color reaction was stopped before epithelial staining began to obscure the mesenchymal expression domain. (**C–N**) Pax9 immunostaining of taste papillae during development on cross sections (C–F; K–N) and horizontal sections of the tongue (G–J). (**C–F**) Pax9 is expressed in the epithelium during CVP morphogenesis and is down-regulated in some regions of the trenches at E18.5 (arrowhead in F). (**G–J**) In addition to the epithelium, Pax9 is also expressed in the mesenchyme during FOP development, while reduced Pax9 levels were observed in the trenches at E18.5 (arrowhead in J). (**K–N**) In the anterior part of the tongue Pax9 is expressed in the FUP epithelium and in filiform papillae (FIP). Note that the expression is very weak or absent in the taste placodes (arrowheads). Scale bars: 200 µm in B; 50 µm in other panels.

Taste buds consist of a group of clustered sensory cells and have been identified in all vertebrates. In the mammalian tongue, taste buds develop in different types of taste papillae: in fungiform papillae (FUP) distributed over the anterior dorsum of the tongue, in circumvallate papillae (CVP) located medially at the back of the tongue, and in foliate papillae (FOP) located laterally at the back of the tongue (Figure 1A). In addition, taste buds form locally without associated papilla structures in the epithelium of the soft palate, throat, epiglottis and upper esophagus. Despite phyletic variations and different distribution patterns of taste papillae, taste buds in the dorsal tongue epithelium develop in all vertebrates, including amphibia, reptiles, birds and mammals. In contrast, larger taste papillae with higher morphological complexity such as the CVP and FOP evolved exclusively in the mammalian lineage [1], [2].

Embryonic induction and development of taste buds have been widely studied in amphibia and rodents (for a recent review, see [3]). These investigations concentrated mainly on the FUPs of mice and rats, which contain taste buds with taste pores that open directly into the oral cavity. FUP development starts around embryonic day 12.5 (E12.5) and involves the formation of an array of epithelial placodes in the anterior two thirds of the tongue. The early patterning of FUP development is regulated by complex signaling processes and involves interactions between the Wnt/β-catenin, Shh and Bmp pathways ([4]–[9]. In mice, each of approximately a total of 90 FUP contains a single taste bud, whereas in some mouse strains the single CVP may house more than 300 taste buds [10], which are located in epithelial trenches that begin to grow into the underlying mesenchyme at E14.5. In addition, small salivary glands (von Ebner's glands) develop together with the CVP and FOP [11] to facilitate gustatory sensation in taste buds located deep in the trenches. Thus, while taste buds of the FUP are formed by epithelial placodes that are established early in development, the placodes of the CVP and FOP undergo substantial morphological changes and intercalate a period of extensive epithelial growth to generate increased taste bud progenitor fields prior to the induction of taste bud cells.

Whereas the CVP and FOP of mammals house the vast majority of taste buds, our understanding of the genetic control of their morphogenesis is surprisingly fragmentary. A single trench was found to develop in the CVP of *Tabby* mice, which lack ectodysplasin A [12], [13]. A CVP placode is missing altogether in mice lacking a functional *Fgf10* gene, which is expressed in the mesenchyme at the pre-placodal stage of CVP development [14]. A malformed CVP or reduction of CVP taste bud number has been described in mice lacking Dystonin, which show insufficient innervation caused by impaired development of the glossopharyngeal cranial nerve [15], as well as in mouse mutants that are compromised in attracting nerve endings due to missing expression of neurotrophins in the CVP epithelium [16]. A recent study revealed a role for *Six1* and *Six4* in CVP development, however, the morphological abnormalities may partly result from defects during cranial nerve formation, which are seen in *Six1*/*Six4*-deficient mice [17]. Thus there are considerable gaps in our knowledge about the developmental mechanisms that regulate the expansion of the early taste bud progenitor cell population in the CVP and FOP epithelium.

The paralogous genes *Pax9* and *Pax1* evolved from a single ancestral gene in the vertebrate lineage and form a subgroup within a total of nine members of the Pax gene family. *Pax9* and *Pax1* regulate different aspects of thymus, skeletal and craniofacial development [18]–[22]. Pax genes encode transcription factors and regulate the morphogenesis of a wide range of organs and are key factors for the development of mammalian sensory organs such as the eye (*Pax6*, *Pax2*), nose (*Pax6*) and ears (*Pax2*, *Pax8*) (for reviews, see [23], [24]). Here we show that *Pax9*, previously not implicated in the development of sensory organs, regulates essential steps during the development of endoderm-derived taste papillae.

## Results

### CVP and FOP development is arrested in *Pax9*-deficient mice

Epithelial expression of *Pax9* in the developing oral apparatus of mice has been documented in the anterior foregut endoderm and its derivatives, as well as in the dorsal epithelium of the tongue [18], [25]. Whole mount X-Gal staining of a developing *Pax9^+/LacZ^* [21] mouse tongue at embryonic day 13.5 (E13.5) indicated that strong Pax9 expression is associated with the localization of placodes forming the CVP and FOP, respectively (Figure 1B). Immunostaining revealed Pax9 expression in the epithelium of placodes and trenches throughout the embryonic period of CVP and FOP development (Figure 1C–J). Pax9 was expressed normally in the region of the developing CVP of E13.5 and E14.5 mouse embryos lacking Fgf10 (Figure S1), a growth factor secreted by the posterior tongue mesenchyme and essential inducer of CVP development [14]. In contrast to the CVP, Pax9 is also expressed in the mesenchyme underlying the FOP epithelium (Figure 1G–J) in cells that are part of two discrete mesenchymal Pax9 expression domains at each side of the tongue (arrowheads in Figure 1B). The expression of Pax9 was down-regulated in some domains of the epithelial trenches at E18.5 (Figure 1F, J), a stage that precedes the early phase of taste bud induction in these papillae. Interestingly, while epithelial cells of the dorsal tongue were also stained, the central regions of placodes forming the FUP were negative for Pax9 at all stages of embryonic development (Figure 1K–N).

**Figure 2:**
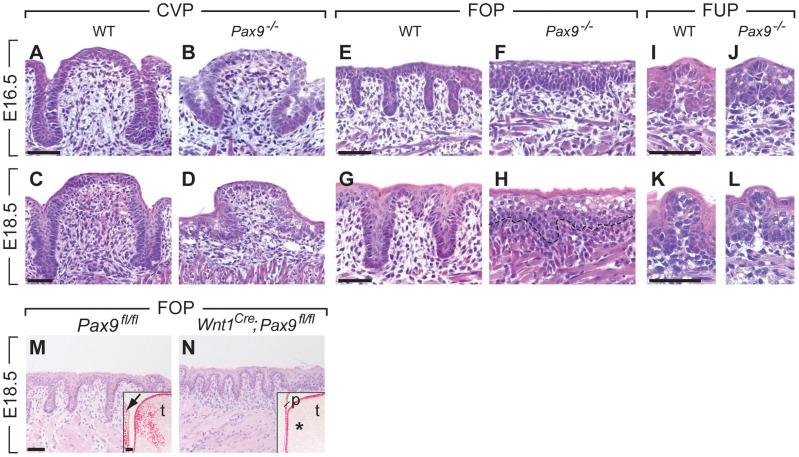
Arrest of CVP and FOP development in *Pax9*-deficient mouse embryos. (**A,C**) In wild type (WT) embryos, the invaginating CVP epithelium forms deep trenches. (**B,D**) Rudimentary CVP trenches form in *Pax9^−/−^* embryos at E16.5 (B) but these trenches fail to invaginate (D). (**E,G**) A series of invaginations develop in the FOP of wild type embryos. (**F,H**) FOP trenches are absent in *Pax9* mutants. (**I–L**) FUP development on the dorsal tongue. The FUP of wild type embryos (I,K) and *Pax9^−/−^* embryos (J,L) are morphologically indistinguishable. (**M,N**) FOP development in *Pax9^fl/fl^* embryos. (M) Without *Cre* expression, FOP development at E14.5 is normal and Pax9 expression is detectable in both epithelium and mesenchyme of the tongue (t), as well as in the adjacent lower jaw mesenchyme (arrow; inset shows a coronal section of the posterior region of the tongue). (N) *Wnt1^Cre^*-mediated inactivation of *Pax9^fl/fl^* did not disrupt the formation of epithelial invaginations. Note that Pax9-positive cells are not detectable in the tongue mesenchyme (asterisk in inset) or in the mesenchyme of the non-elevated secondary palate (p). Scale bars: 50 µm.

A histological analysis of serial sections of the three taste papilla types developing in the mouse tongue revealed that *Pax9* is required for the formation of epithelial invaginations in both CVP and FOP. In homozygous *Pax9^LacZ/LacZ^* (for simplicity referred to as *Pax9^−/−^* hereafter) embryos, a CVP placode forms (Figure S2) but the epithelial trenches are growth retarded at E16.5 and E18.5 (Figure 2A–D). Similarly, invaginations of the FOP are missing and keratinocytes of the superficial layers are aberrantly enlarged in the mutant epithelium (Figure 2E–H). Moreover, the thickness of the mesenchymal cell layer was greatly reduced in the mutant FOP at E18.5. In contrast, the morphology of FUP appeared normal in *Pax9^−/−^* embryos (Fig. 2I–L).

To address the role of *Pax9* in neural crest cell-derived mesenchymal cells located adjacent to the developing FOP (Figure 1G–J), we inactivated the *Pax9* gene in these cells by crossing *Pax9^flox^* (*Pax9^fl^*) mice [26] with transgenic mice expressing Cre under the control of *Wnt1* promoter (*Wnt1^Cre^*; [27]). While the *Pax9^fl/fl^* alleles were efficiently recombined in *Wnt1^Cre^*;*Pax9^fl/fl^* embryos, mesenchymal cells underlying the FOP were present and epithelial trenches formed in all (n = 5) mutant FOP of *Wnt1^Cre^*;*Pax9^fl/fl^* embryos (Figure 2M, N). These findings indicate that Pax9 function during FOP development is primarily required in epithelial cells.

### *Pax9* is dispensable in the developing and adult FUP

**Figure 3:**
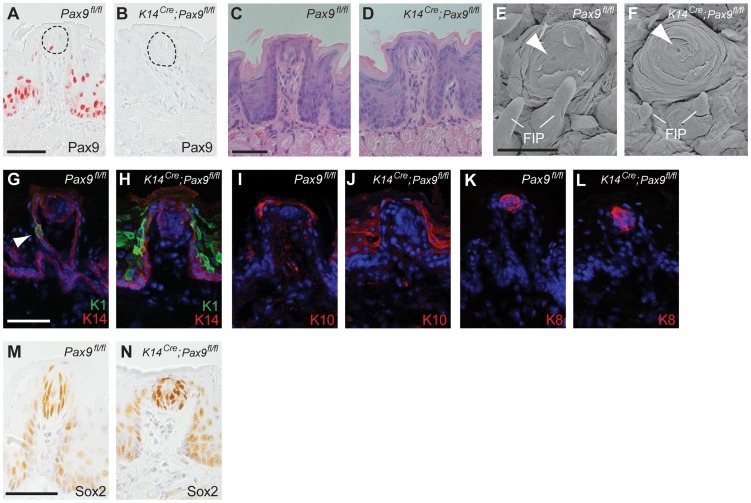
FUP maintenance and FUP taste bud renewal do not require Pax9 functions. All analyses were carried out using 3–5 months old mice. (**A,B**) Pax9 immunostaining of FUPs. In *Pax9^fl/fl^* mice (A), Pax9 expression is detected in the FUP epithelium and in isolated taste bud cells (area of taste bud is indicated by dotted line). (B) No Pax9-positive cells are detectable in the FUP after *K14^Cre^*-mediated recombination of *Pax9^fl/fl^*. (**C,D**) Histological sections of FUP. *Pax9^fl/fl^* FUP (C) and *K14^Cre^*;*Pax9^fl/fl^* FUP (D) are morphologically indistinguishable. (**E,F**) Scanning electron microscopy images of FUP. The FUP of both *Pax9^fl/fl^* (E) and *K14^Cre^*;*Pax9^fl/fl^* (F) form taste pores (arrowhead), whereas the non-sensory FIP of the mutants (F) are hypoplastic. (**G–L**) Indirect immunofluorescent detection of keratins. Nuclei were stained with DAPI (blue). (G) In *Pax9^fl/fl^* mice, K14 is expressed in basal cells of the epithelium and K1 expression was seen in isolated epithelial cells of the FUP epithelium (arrowhead). (H) While K14 expression was not affected in the FUP of *K14^Cre^*;*Pax9^fl/fl^* mice, the number K1 expressing cells was strongly increased. (**I,J**) K10 expression is mainly restricted to the apical end of the FUP in *Pax9^fl/fl^* mice (I) whereas its expression is more extended in *K14^Cre^*;*Pax9^fl/fl^* mice (J). (**K,L**) K8 expression marks taste bud cells in both genotypes. (**M,N**) Immunohistochemical staining showing that Sox2 is expressed in mature taste buds of both *Pax9^fl/fl^* (M) and *K14^Cre^*;*Pax9^fl/fl^* (N) mice. Scale bars: 50 µm in A,C,G,M; 500 µm in E.

Postnatal Pax9 expression continues not only in the FUP epithelium but was also found in a few taste bud cells of the fully differentiated FUP (Figure 3A). Since FUP development is completed postnatally and since taste buds do not form prior to 2 days after birth we asked if *Pax9* could be required at these later stages of FUP development. Because *Pax9^−/−^* embryos die at birth, we addressed this question by using transgenic mice expressing Cre under the control of Keratin 14 (*K14^Cre^*) promoter [28]. Previous studies showed that K14 is expressed in basal cells of the tongue epithelium and in FUP but not in actual taste bud cells. However, lineage tracing experiments identified K14-positive epithelial cells located directly adjacent to the taste bud as a niche of stem cells renewing taste bud cells in the adult mouse [29]. X-Gal staining of *K14^Cre^*;*ROSAR26* embryos confirmed efficient *Cre* activity in the dorsal tongue epithelium from E13.5 onwards (Figure S3A,B) and Pax9 immunostaining revealed complete removal of Pax9 protein in both FUP and its associated taste buds in adult *K14^Cre^*;*Pax9^fl/fl^* mouse tongues (Figure 3B). Interestingly, the size and morphology of adult FUPs was not affected and FUP taste buds in these mutants appeared normal and formed taste pores (Figure 3C–F). In addition, the number of FUP visible on the dorsal aspect of *K14^Cre^*;*Pax9^fl/fl^* mouse tongues (30 per tongue, n = 5) did not differ significantly (p>0.79) from the number of FUPs of control mice (31 per tongue, n = 5).

To characterize the differentiation of the adult, *Pax9*-deficient FUP epithelium, we analyzed the expression of various keratin (K) proteins, which form intermediate filaments in cell type-specific combinations. We found that the keratin pair K1 and K10, which are normally expressed throughout the differentiated suprabasal layers of the epidermis, were strongly up-regulated in the *K14^Cre^*;*Pax9^fl/fl^* FUP epithelium, as well as in the interpapillary epithelium (Figure 3G–J). The expression of K6, which is often seen in hyperproliferative epidermal cells [30], was also up-regulated in the interpapillary epithelium, but not in the FUP epithelium itself (Figure S4A,B). In contrast, the expression of K14 and K5, which are normally found in basal cells of all stratified squamous epithelia, was not changed (Figure 3G,H; Figure S4C,D). Finally, we did not observe changes of the expression of K8, which marks taste bud cells in all taste papillae, as well as of Sox2, a marker of mature taste bud cells and critical regulator for the formation of taste sensory cell [31], in *K14^Cre^*;*Pax9^fl/fl^* mouse tongues (Figure 3K–N). Together, these results indicate that *Pax9* is not functionally involved in the development of the mouse FUP. Furthermore, although the *Pax9*-deficient FUP epithelium shows alterations of keratin expression patterns, these changes are not associated with apparent defects of FUP maturation and FUP maintenance in the adult mouse. In contrast, Pax9 is required for the formation of filiform papillae (FIP), epithelial projections of the dorsal tongue epithelium that do not contain taste buds (Figure 3E,F; [25]).

Unlike the epithelium of the dorsal tongue, we did not detect full *K14^Cre^* activity in the CVP and FOP epithelium during embryonic development (Figure S3A,B). At perinatal stages, *K14^Cre^* activity expands to posterior regions of the tongue (Figure S3C) and while complete inactivation of *Pax9* gradually manifests in the CVP and FOP of *K14^Cre^*;*Pax9^fl/fl^* mice, *Pax9* deficiency was not associated with obvious morphological defects in these taste papillae (Figure S3D,E). In summary, the data indicate that Pax9 functions are not needed in adult taste papillae and that the requirement for Pax9 is restricted to the early steps of CVP and FOP morphogenesis.

### Epithelial differentiation defects of the *Pax9*-deficient CVP are associated with the absence of proneural induction

**Figure 4:**
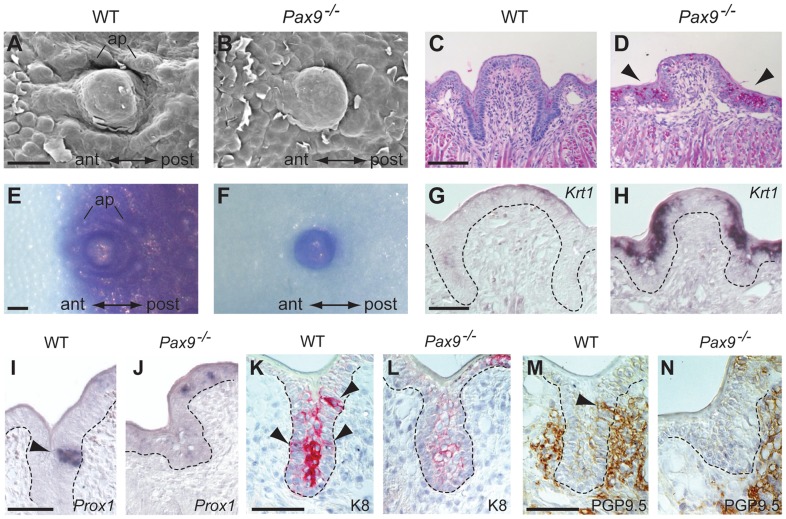
Differentiation defects and lack of proneural induction in the *Pax9*-deficient CVP trench epithelium. (**A–N**) Analyses of mouse embryos at E18.5. Anterior (ant) to posterior (post) orientation is indicated where appropriate. (**A,B**) SEM images of the CVP showing that both central dome and accessory papillae (ap) are well developed and separated by trenches in the wild type (A) but not in the *Pax9^−/−^* embryo (B). (**C,D**) PAS staining indicates intensively increased concentration of mucopolysaccharides in the mutant CVP trenches (arrowheads in (D). (**E,F**) Whole-mount barrier assay revealing that the CVP and the posterior tongue epithelium is permeable to toluidine blue in the wild type embryo (E), while a premature barrier has formed in the epithelium surrounding the mutant CVP (F). (**G,H**) *Krt1* RNA in situ hybridization showing that *Krt1* expression is strongly up-regulated in the *Pax9^−/−^* CVP (H). Dashed lines indicate the margin of the trench epithelium. (**I,J**) In situ hybridization of *Prox1*. Groups of epithelial trench cells express the proneural marker *Prox1* in the wild type (I) but not in the *Pax9^−/−^* embryo (J). (**K,L**) Immunostaining of K8. Similar to *Prox1*, K8 is locally expressed in the wild type CVP (arrowheads in K). In contrast, only weak expression of K8 was detectable in the mutant CVP (L). (**M,N**) Immunostaining of PGP9.5. In the wild type CVP (M), nerve fibers contact the CVP trench epithelium (arrowhead; this section is directly adjacent to that shown in (K)), while nerve endings fail to invade the CVP trench epithelium of the *Pax9^−/−^* embryo (N). Scale bars: 100 µm in A,C,E; 50 µm in G,I,K,M.

While the FOP of *Pax9^−/−^* mutants does not form any epithelial trenches, the CVP exhibits rudimentary invaginations (Figure 2) and we thus chose the latter to characterize the cellular and molecular defects during embryonic CVP morphogenesis. SEM of the posterior tongue region showed that newborn *Pax9^−/−^* mice lack an accumulation of accessory papillae that normally surround the central domain of the CVP (Figure 4A,B). We also noted increased desquamation of the posterior tongue epithelium and diastase-controlled PAS staining revealed strongly increased levels of glycogen in the area in which the CVP trenches normally develop (Figure 4C,D). This differentiation defect is reminiscent of inappropriately increased deposition of glycogen regularly observed in the benign condition glycogenic acanthosis of the esophageal epithelium [32]. Moreover, a barrier assay revealed that only the central domain of the *Pax9*-deficient CVP was permeable to toluidine blue solution at E18.5, whereas the surrounding mutant tongue epithelium has prematurely established a full barrier (Figure 4E,F). Furthermore, the mutant CVP epithelium expresses high levels of *Krt1* (Figure 4G,H), a keratin gene that is normally expressed in the mouse skin and not in the tongue [25] but was found to be up-regulated in oral dysplasia [33]. Together, these findings document the inappropriate differentiation of the *Pax9*-deficient CVP epithelium.

During mouse CVP development, taste buds become morphologically distinct from the surrounding trench epithelium two days after birth. Thus, to visualize epithelial domains that have started to initiate taste bud formation in the CVP at E18.5, we analyzed the expression of K8 and *Prox1*, which both mark taste bud primordia at this developmental stage [34], [35]. Both markers identified groups of cells in wild type epithelial trenches but not in the trenches of *Pax9*-deficient mice (Figure 4I–L). The same result was found using an *Ascl1* (previously called *Mash1*) probe for in situ hybridization (Figure S5). In addition, K8 expression, normally found in loosely aligned cells in the middle of each trench (Figure 4K), was strongly reduced in the mutant CVP (Figure 4L). Interestingly, expression of *Prox1* and K8 was also found in the apical domain of the CVP in both wild type (Figure S6A,B) and *Pax9*-deficient mice at E18.5 (Figure 4J,L; Figure S6C). We did not further investigate these structures, which are likely to represent immature taste buds that lack taste pores [36] and are known to disappear at early postnatal stages [37].

Afferent nerve fiber endings of the glossopharyngeal nerve make contact with the CVP epithelium from E14.5 onwards [38]. To analyze the pattern of CVP innervation, we stained for the neural marker PGP9.5 [39], which revealed a close contact of nerve fibers with the trench epithelium of the wild type CVP (Figure 4M). In contrast, although branches of the glossopharyngeal nerve were present at the *Pax9*-deficient CVP, we did not detect any penetration of the mutant trench epithelium by nerve endings (Figure 4N).

### Disruption of the Shh signaling pathway in *Pax9*-deficient CVP and FOP

**Figure 5:**
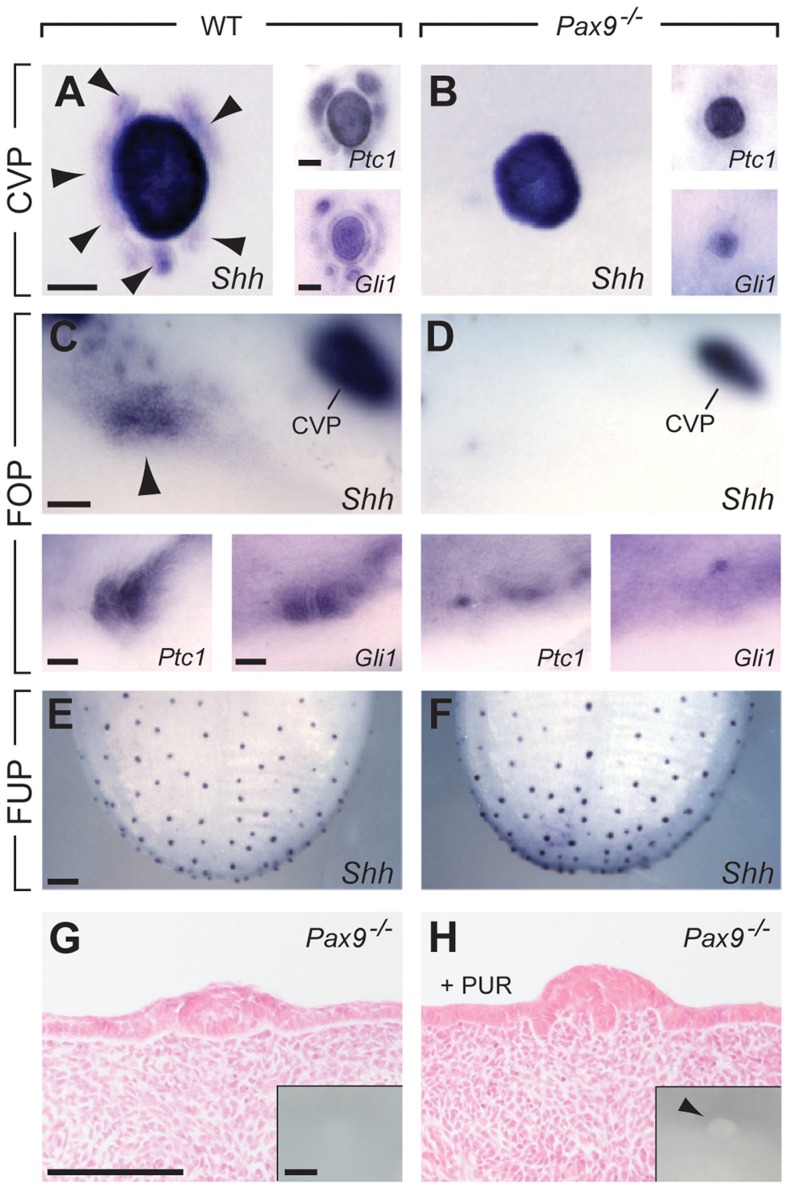
Absence of *Pax9* causes an endoderm-specific disruption of the Shh pathway in taste papillae. (**A–F**) Whole mount in situ hybridization of *Shh*, its receptor (*Ptc1*) and the downstream effector transcription factor (*Gli1*) at E14.5. (**A,B**) In the wild type CVP (A), *Shh* is expressed in the central dome as well as in a ring of accessory papillae (arrowheads). *Ptc1* and *Gli* are expressed in a similar pattern. In the absence of *Pax9*, *Shh*, *Ptc1* and *Gli1* are only expressed in the central dome of the CVP (B). (**C,D**) In wild type embryos (C), patches of *Shh*, *Ptc1* and *Gli1* expression are detectable in the region of the developing FOP, whereas these expression patterns are missing (*Shh*) or are greatly reduced (*Ptc1*, *Gli1*) in *Pax9^−/−^* embryos (D). (**E,F**) *Shh* expression in FUP placodes is similar in wild type (E) and *Pax9*-deficient (F) embryos. (**G, H**) Histological sections of *Pax9*-deficient, cultured embryonic tongues. (G) In control medium the *Pax9^−/−^* CVP of cultured tongues is small and is not visible externally (inset). (H) In the presence of purmorphamine (PUR) the number of epithelial cells is increased in the dome of the CVP. Note the absence of trenches. Inset shows enlarged, protruded CVP dome (arrowhead) of the cultured tongue. Scale bars: 100 µm in A,C,G: 200 µm in E.

The Shh signaling pathway is active in taste papillae of the developing mouse tongue [40] and its inhibition was found to increase the number of FUP in the dorsal tongue epithelium [6], [7]. At the early stage of CVP development (E13.5), we found *Shh* expression in the epithelial placode in both control and *Pax9*-deficient embryos (Figure S2). At E14.5, in addition to the central, dome-like structure of the CVP, a ring of accessory papillae surrounding the center of the CVP was *Shh*-positive in controls, but not in *Pax9^−/−^* embryos (Figure 5A,B). Similar patterns were obtained with probes for the Shh pathway downstream genes *Ptch1* and *Gli1*, in addition to a strong down-regulation of *Gli1* expression in the center of the mutant CVP (Figure 5A,B). In the developing FOP, *Shh* expression was considerably weaker compared to that of the CVP but expression in an indistinctly delimited area was consistently identified on both sides in the posterior part of the wild type tongue (Figure 5C). In *Pax9^−/−^* mutants, *Shh* expression levels were below the detection threshold and only very weak expression of *Ptch1* and *Gli1* was found (Figure 5D). In contrast, consistent with unaffected FUP development, *Shh* was normally expressed in the dorsal tongue epithelium of *Pax9^−/−^* mutants (Figure 5E, F).

Since the Shh pathway is an important modulator of epithelial morphogenesis during the development of various ectodermal appendages [41]–[43] we speculated that a reduction of Shh pathway activity in the developing CVP could be related to the impaired growth of the trenches in *Pax9^−/−^* embryos. To test this, we cultured mutant embryonic tongues in the presence of purmorphamine, a Shh signaling agonist that targets the Shh pathway effector protein Smoothened [44]. Under culture conditions used in this study, embryonic tongues dissected at E13.5 and cultured for 48 hours in control medium either formed a small CVP or an epithelial bud. In the presence of purmorphamine, the size of the mutant CVP (n = 4) was significantly increased but growth was primarily stimulated in the central, dome-like domain of the CVP (3 out of 4, Figure 5H). A similar response was observed in *Pax9-*deficient tongues cultured in the presence of a Shh protein-loaded bead placed next to the CVP. However, this result was only seen when the Shh protein-loaded bead was not displaced during culture (Figure S7A,B). In contrast, an enlarged CVP dome or enhanced trench formation was not observed after purmorphamine treatment of explants from wild type embryos (Figure S7C).

### *Pax1* is a critical target of *Pax9* in the proliferating CVP trench epithelium

**Figure 6:**
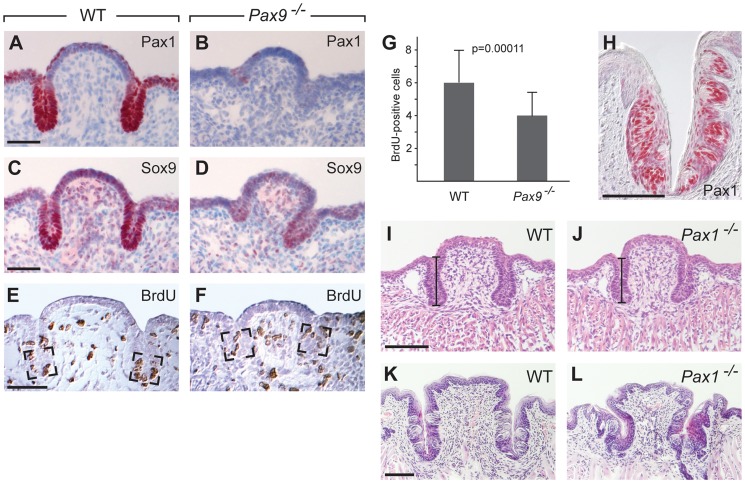
*Pax1* and *Sox9* are *Pax9* targets in the proliferating compartment of the CVP trenches. (**A–F**) Immunohistochemical staining on sections of the CVP at E15.5. (**A,B**) Pax1 is strongly expressed in the tips of epithelial trenches and in periderm cells covering the central dome of the wild type CVP (A), but not in the *Pax9*-deficient CVP (B). (**C,D**) Similarly, Sox9 expression is strongest in the epithelial trenches (C) and is barely detectable in the *Pax9* mutant CVP (D). (**E,F**) BrdU-positive cells were counted in defined areas (boxed) of the CVP trenches from three wild type (n = 29 sections) and three *Pax9* mutant CVPs (n = 28 sections). (**G**) The number of proliferating cells in the *Pax9*-deficient CVP is significantly reduced. Error bars illustrate standard deviation. (**H**) Pax1 immunostaining of one CVP trench in a 3 months old wild type mouse. (**I,J**) Morphology of the CVP at E18.5. The lengths of the CVP trenches (indicated by bars) were measured and shown to be reduced in the absence of *Pax1* (for summary of measurements see Table S1). (**K,L**) Morphology of the CVP at postnatal day 16. In *Pax1* mutants (n = 3) the trenches are growth-retarded and contain fewer taste buds. Scale bars: 50 µm in A,C,E; 100 µm in H,I,K.

The incomplete ability of Shh pathway activation to restore epithelial growth of the *Pax9*-deficient CVP prompted us to search for additional developmental pathways that may be affected in the CVP of *Pax9^−/−^* mutants. To screen for early molecular defects, a genome-wide RNA expression analysis of wild type and *Pax9*-deficient CVP dissected at E14.5 was carried out. The array data suggested that two genes encoding the transcriptional regulators Sox9 and Pax1 might present early targets of Pax9 in the developing CVP. Immunostaining indeed confirmed that both transcription factors are strongly expressed at the tips of invaginating trenches of the normal CVP, but not in the CVP of *Pax9^−/−^* mutants (Figure 6A–D). Sox9 and Pax1 were shown to regulate epithelial cell proliferation in various developmental systems [45]–[47] and in agreement with these functions, counting of BrdU-positive cells at the tip of the growing trenches revealed a significant reduction of the number of proliferating cells in *Pax9^−/−^* mutants (Figure 6E–G).

*Pax1* and *Pax9* are paralogous genes and while they have redundant functions during vertebral column development [22], the absence of Pax1 expression in the *Pax9*-deficient CVP rules out that Pax1 may compensate for the loss of Pax9 during early CVP development. Interestingly, Pax1 itself continues to be expressed and labels most taste bud cells in the wild type CVP and FOP of adult mice (Figure 6H; Figure S8A). In contrast, Pax1 is not expressed in the dorsal tongue epithelium during FUP development or in the FUP of adult mice (Figure S8B,C). Analysis of mouse mutants with a targeted deletion of *Pax1* [48] showed that they develop shorter CVP trenches at E18.5 (111 µm in *Pax1* ^−/−^ mutants, 131 µm in control littermates, n = 8, p<0.01), while the width of the CVP was not significantly changed (Figure 6I,J; Table S1). Histological analysis of the CVP at postnatal day 16 revealed that the CVP of *Pax1*-deficient mice was noticeably smaller (n = 3; Figure 6K,L). Corresponding with this growth retardation, counting taste buds of a complete series of sections of one *Pax1* mutant CVP indicated that the total number of taste buds was reduced by more than 50%. Thus *Pax1* expression in the CVP trenches is required for epithelial growth and for the generation of the normal number of taste buds in the mouse CVP.

### Taste placodes in the soft palate are missing in *Pax9^−/−^* mutants

**Figure 7:**
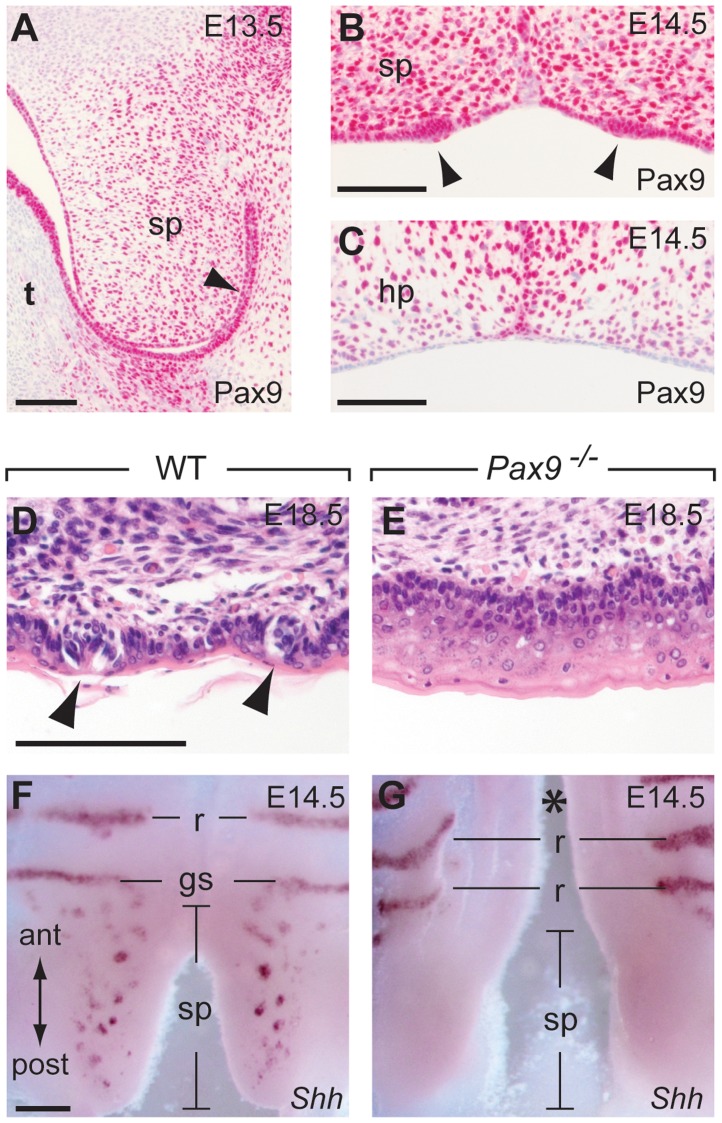
Pax9 is essential for taste placode formation in the soft palate. (**A–C**) Pax9 immunostaining of the secondary palate. (**A**) At E13.5, Pax9 expression is found in the mesenchyme as well as in those epithelial cells (arrowhead) of the soft palate (sp) facing the oral cavity after palatal shelf elevation. (**B**) At E14.5 the palatal shelves have elevated and Pax9 expression is seen in epithelial placodes (arrowheads) of the soft palate. (**C**) Pax9 is not expressed in the epithelium of the hard palate (hp). (**D,E**) Histological staining revealed taste bud precursors in wild type (D), but not in the *Pax9*-deficient (E) epithelium of the soft palate at E18.5. (**F,G**) Whole-mount *Shh* in situ hybridization at E14.5. In the wild type soft palate (F), *Shh* expression marks the taste placodes of the soft palate as well as the “Geschmacksstreifen” (gs). Note that palatal rugae (r) also express *Shh* at this stage. (G) *Shh* expression is not detectable in the soft palate of *Pax9* mutants, which also form a cleft secondary palate (asterisk). Scale bars: 100 µm in A–D; 200 µm in F.

The posterior part of the secondary palate forms the soft palate which, in contrast to the hard palate, is movable and not supported by bones. Moreover, the oral mucosa of the soft palate is part of the gustatory system and forms taste buds, however, these taste buds lack supporting papilla structures and are directly embedded in the epithelium (Figure 7D). During soft palate development, Pax9 expression was detected in the mesenchyme as well as in the epithelium prior to palatal shelf elevation (Figure 7A). Taste placodes of the soft palate begin to form as epithelial thickenings at E14.5 and express *Shh* [49]. Both taste placodes and soft palate epithelium are Pax9-positive, whereas Pax9 is not expressed in the epithelium of the hard palate, which lacks these placodes (Figure 7B,C). In newborn *Pax9^−/−^* mice, no clusters of taste bud progenitors were found in the soft palate epithelium (Figure 7D,E) and complete absence of *Shh* expression at E14.5 indicates that taste placode induction is not initiated in the soft palate of *Pax9^−/−^* mutants (Figure 7F,G).

## Discussion

Taste perception at the back of the mammalian oral cavity serves as a critically important control mechanism to discriminate nutritious ingredients from substances that are potentially toxic to the organism. The formation of epithelial trenches that are rinsed by saliva produced in associated minor salivary glands enables a high concentration of functional taste buds to form in the narrow, posterior part of the tongue. The complex architecture of the CVP and FOP, and the close vicinity of numerous taste buds in these taste papillae predict the activities of developmental programs to differ from those regulating patterning and development of the FUP on the anterior dorsal tongue. Indeed, while loss of Fgf10 signaling in the mouse tongue mesenchyme results in the absence of the CVP, the spacing and size of FUP increased in these mutants [14]. In addition, similar to the differential expression of Pax1 shown in this work, expression of a Bmp4 reporter allele was detected in taste buds of the CVP but not in taste buds of FUP [50]. These fundamental differences may be attributed to the different embryonic origins of various taste papillae. Strong support for an entirely endoderm-derived origin of the CVP and FOP was recently provided by lineage tracing of Sox17-2AiCre/R26R mouse embryos [51],[52]. The study also indicated that the FUP on the dorsal tongue are exclusively derived from ectodermal cells.

During the development of the oral epithelium, expression of Pax9 is not restricted to endoderm-derived structures but is also seen in ectoderm-derived FUP as well as in non-sensory filiform papillae (this work; [25]). Our results clearly demonstrate that *Pax9*-deficiency does not affect patterning, development or maintenance of the mouse FUP. Although this result was unexpected, it reinforces the conclusion that endoderm-specific developmental pathways regulate the formation of the gustatory system in the posterior region of the oral cavity.

The early steps of CVP morphogenesis follow a sequence that is similar to that typically seen during the development of organs which form by epithelial-mesenchymal interactions. In analogy to the formation of, for example, a mammalian tooth or hair follicle, the CVP placode forms a bud-like epithelial structure that subsequently branches to form lateral invaginations. While the initial branching of the CVP bud is not affected in *Pax9* mutant embryos, subsequent invagination of the epithelial trenches is blocked. Interestingly, a characteristic ring of accessory papillae normally surrounding the central dome of the CVP was not established in *Pax9*-deficient embryos. Whereas the developmental role of the accessory papillae has not been studied thus far, we found that they express *Shh*, suggesting that they may function as transient signaling centers and thereby contribute to CVP morphogenesis. The mitogenic effect of Shh has been documented in various epithelia [53]–[55] and we here found that activation of the Shh downstream pathway by purmorphamine increased the size of the *Pax9*-deficient CVP in embryonic tongue cultures. However, epithelial trench formation could not be rescued in these experiments, raising the possibility that precise timing and localization of Shh secretion by accessory papilla cells are required to restrict cell proliferation to the rudimentary trenches. Inhibition of the Shh pathway in rat embryonic tongue cultures was shown to increase the number of FUP [6]. While the external morphology of the CVP was not altered by Shh pathway inhibition, formation and growth of the epithelial trenches was not analyzed in these experiments. Recently, mouse reporter strains mapping the expression of the Shh pathway and its downstream genes in embryonic and adult FUP convincingly demonstrated an association between *Shh* expression and proliferation in neighboring epithelial cells [29], [56]. Thus, *in vivo* experiments using genetic tools suitable to inactivate or activate the Shh pathway in the CVP in an inducible manner should help to identify the specific roles of Shh for patterning and morphogenesis during CVP development.

Our analyses identified Pax9 as the first developmental regulator that is directly required for the expansion of taste progenitor cells in the developing mouse CVP. This progenitor field is normally established during a period of epithelial growth between E14.5 and E18.5 and our BrdU-labeling revealed a high proportion of cells that proliferate at the tip of the CVP trenches. Proliferation is significantly reduced in the invaginating epithelial CVP trench cells of *Pax9* ^−/−^ embryos, and this cellular defect is associated with a drastic down-regulation of Sox9, a known regulator of epithelial cell proliferation in other developing organs [45], [46], [57]. Beside this, Sox9 is necessary to establish the stem cell compartment in the hair follicle [58], raising the possibility that Sox9 could have a similar function in the CVP.

*Pax1* and *Pax9* exhibit similar expression patterns during embryonic development and function in a redundant manner during the formation of the vertebral column [18], [22]. Similarly, *Pax1* and *Pax9* both regulate aspects during the development of the thymus, which is derived from the foregut endoderm [20], [21]. Interestingly, while *Pax1* is more critically required during vertebral column development, the role of *Pax9* is more important in foregut-derived organs, to which the expression of the common *Pax9/1* precursor is restricted in early chordates [59]. The moderate CVP phenotype of *Pax1^−/−^* mice identified in this work appears to support this conclusion. Together, these findings suggest that the mammalian *Pax9* gene has retained the original function of the common *Pax9/1* precursor gene in the foregut endoderm, while *Pax1* has acquired a predominant role in the axial skeleton during vertebrate evolution.

Besides their functions in taste papilla formation, the expression of Pax9 and Pax1 in taste buds of adult mice suggests additional roles in the fully matured gustatory system. The absence of isolated, Pax9-positive cells in FUP taste buds after *K14Cre*-mediated recombination did not cause obvious morphological defects of the taste buds. However, as *K14Cre* is not active in actual taste bud cells, this finding supports the conclusion that stem cells from adjacent, non-sensory FUP cells contribute to the renewal of FUP taste buds [29]. While the roles of Pax9 and Pax1 in taste buds remain to be elucidated using appropriate genetic tools, it is tempting to speculate that they could be involved in the specification of sub-populations of mature taste bud cells.

Absent expression of K8 and *Prox1* and lack of contact by nerve endings in the developing CVP trenches, as well as premature barrier formation indicates a highly defective differentiation program of the posterior tongue epithelium of *Pax9*-deficient embryos. In the mutants, the arrest of CVP morphogenesis is associated with ectopic expression of *Krt1*, a keratin gene known to be strongly up-regulated in dysplasia of the oral epithelium [33], as well as with increased levels of glycogen, a feature seen in the benign condition glycogenic acanthosis [32]. It therefore appears likely that premature and inappropriate terminal differentiation of the CVP epithelium accounts, at least in part, for the incompetence of the CVP trench cells to interact with nerve fiber endings and to generate taste bud progenitors.

Our data show that epithelial trench formation in the CVP and FOP is *Pax9*-dependent. A primary function for *Pax9* in the expansion of taste progenitor fields in taste papillae with a higher degree of architectural complexity appears to be supported by the finding that taste papillae on the dorsal tongue, which lack epithelial trenches, develop normally in *Pax9*-deficient mice. However, although the soft palate epithelium does normally not form any recognizeable taste papilla structures, *Pax9* is required for early *Shh* expression and for the induction of taste progenitor cells in this part of the oral cavity. Interestingly, lack of *Shh* expression in the taste placodes of the soft palate was also observed in mouse mutants lacking β-Catenin in the epithelium [60], raising the possibility that *Pax9* might interact with Wnt-signalling. A complete secondary palate only evolved in the mammalian lineage, whereas the tongue is present in amphibia, reptiles, birds, and mammals [1]. While the molecular mechanisms regulated by *Pax9* in the soft palate epithelium remain to be identified, it is conceivable that *Pax9* may have acquired an additional, early role for taste placode formation in the soft palate epithelium at a later period during the evolution of tetrapods.

## Materials and Methods

### Ethics statement

All procedures were carried out under personal and project licenses issued by the Home Office, UK and were approved by the Local Ethics Committee.

### Mouse husbandry and genotyping

Mice were housed as described previously [61]. Embryos were staged by taking mid-day on the day of vaginal plug detection as embryonic day 0.5 (E0.5). The following mouse lines were maintained on the indicated genetic background, intercrossed to produce relevant genotypes and PCR genotyped according to references: Pax9^lacZ^ (C57BL/6; [21]), Pax9^flox^ (C57BL/6 x 129S2/SvPas; [26]), Wnt1^Cre^ (C57BL/6; [26]), K14^Cre^ (FVB/N; [28]), Pax1 (C57BL/6; [48]), ROSA26R (C57BL/6; [62]).

### Histology and immunohistochemistry

Mouse tissues were prepared, processed, paraffin-embedded, sectioned, stained with haematoxylin and eosin and photographically documented as described previously [61]. Diastase-controlled Periodic acid-Schiff (D-PAS) staining was performed as described [63]. CVP size was measured using AxioVision software v.4.3 (Carl Zeiss) and statistically analyzed by a two-tailed t-test (Excel software, Microsoft).

Pax9 immunohistochemical staining on paraffin sections was performed as described previously [64] with the following modifications. Antibodies were diluted in antibody diluent (Dako, S3022) and incubated in the following order: rat anti-Pax9 (1∶40), rabbit anti-rat IgG (Dako, Z0494; 1∶50), rat APAAP (Dako, D0488; 1∶50) with three TBS washes in between each step. The last two steps were repeated and alkaline phosphatase activity was visualized using Fast Red (Sigma) as a substrate. Other primary antibodies were detected using the Envision+ System-HRP kit (Dako, K4008 or K4010) according to the manufacturer's instructions. AEC (Dako, K4008) and DAB (Dako, K4010) substrates stain red and brown, respectively. Primary antibodies were used at the following dilutions: rabbit anti-PGP9.5 (7863-0504, AbD Serotec), 1∶200; rabbit anti-Sox2 (C70B1, Cell Signaling), 1∶100; rabbit anti-Sox9 (O9-1, [65]), 1∶1000; rat anti-Pax1 [66], 1∶40. Following incubation with rat anti-Pax1 antibody, HRP-conjugated rabbit anti-rat IgGs (Dako, P0450) were applied at 1∶200 dilution before using the rabbit-specific Envision+ detection system.

To visualize proliferating cells, BrdU labeling and detection was performed as described previously [67]. Serial sections from three wild type (29 sections) and three *Pax9*-deficient (28 sections) E15.5 CVPs dissected 90 minutes after BrdU injection were prepared and BrdU-positive cells counted in a defined area at the tip of epithelial trenches. Statistical significance was assessed using a two-tailed t-test.

For indirect immunofluorescence analysis, 5 µm cryosections were air-dried on Superfrost ultra plus slides (Thermo Scientific) for 2 hours at room temperature and then fixed for 10 minutes with pre-cooled acetone at −20°C. Immunofluorescence analysis was performed as previously described [68], using the following primary antibodies: rabbit anti-K1 (AF109, Covance), 1∶1000; mouse anti-K10 (DE-K10, Progen), 1∶160; rabbit anti-K5 (Covance), 1∶5000; guinea pig anti-K14 (GPCK14.2, Progen), 1∶50; mouse anti-K6 (Ks6.Ka12, Progen), 1∶10; rat anti-K8 (TROMA-I, Developmental Studies Hybridoma Bank), 1∶50. Nuclei were stained with DAPI (Invitrogen) and secondary antibodies were species-specific fluorochrome-conjugated goat antibodies: Cy3-conjugated anti-mouse and anti-guinea pig, both 1∶200; Alexa 594-conjugated anti-rat and Alexa 488-conjugated anti-rabbit, both 1∶400 (Molecular Probes). Microscopic analysis was performed using a Leica SP2 UV confocal microscope operated through LCS 2.61 software (Leica Microsystems).

### Barrier assay, X-Gal staining and scanning electron microscopy (SEM)

Tongue barrier assays and whole-mount X-gal staining were performed as described previously [25], [21]. For SEM, tongues were fixed in 2% glutaraldehyde/PBS, dehydrated through a graded series of ethanol followed by carbon dioxide incubation in a Samdri 780 Critical Point Dryer. The specimens were then mounted on an aluminium stub with Acheson Silver Electrodag (Agar Scientific) and coated with gold using a Polaron SEM coating unit. Specimens were examined and photographed using a Stereoscan 240 scanning electron microscope. SEM images taken from flat-mounted tongues of 4 months old mice were also used to count the number of FUPs that were directly visible on the dorsal tongue surface.

### RNA in situ hybridization

RNA in situ hybridization of whole embryonic specimens and of tissue sections using digoxygenin-labelled cRNA probes was performed as described previously [67]. cRNA probes were produced for *Shh* (0.6 kb; MGI:1327804), *Ptch1* (2.2 kb; MGI:3833867), *Gli1* (1.7 kb; MGI:12533), *Prox1* (0.5 kb; [69]), *Ascl1* (0.7 kb; [69]), and *Krt1* (0.5 kb; [25]).

### Embryonic tongue culture

Embryonic mandibles including tongues were dissected at E13.0 and cultured for two days as described previously [70], [4]. Before culture, the specimens were embedded in growth factor-reduced Matrigel (BD Biosciences, Cat. No. 305128) to prevent them from flattening during culture. To activate the Hh pathway, 4 µM purmorphamine (Calbiochem, Cat.No. 540220) was added to the culture medium. Alternatively, Affi-Gel Blue gel beads (Bio Rad, Cat.No. 153-7302) were soaked in recombinant mouse SHH protein (1.25 mg/mL in PBS; R&D Systems, Cat.No. 461-SH) or BSA for at least an hour and the beads were then placed onto the tongue epithelium close to the developing CVP.

## Supporting Information

Figure S1 Immunohistochemical staining of Pax9 in the posterior tongue epithelium of *Fgf10*-deficient mouse embryos. At E13.5 (A) and E14.5 (B) Pax9 is expressed in epithelial cells of the tongue region in which the CVP normally develops. Scale bar: 50 µm.
(TIF)

Figure S2 *Shh* expression in the CVP placode at E13.5. In situ hybridisation on sections showed that *Shh* is expressed in the early CVP epithelium of both wild type (A) and *Pax9* mutant (B) embryos. Dotted lines outline the border between epithelium and mesenchyme and insets show *Shh* expression by whole mount in situ hybridisation. Scale bars: 50 µm.
(TIF)

Figure S3 (**A–C**) X-Gal staining of *K14^Cre^;ROSA26R* mouse embryonic tongues at E13.5 (A), E14.5 (B), and P0 (C). Note absence of *K14^Cre^* activity in the posterior region of the tongue (arrowheads) at embryonic stages. (**D,E**) Pax9 immunostaining of CVP (D) and FOP (E) in adult *K14^Cre^;Pax9^fl/fl^* mice. Although little (CVP) or no (FOP) Pax9 protein is detectable, the morphology of the taste papillae and taste buds appears normal. Inset shows Pax9 staining in one of the minor salivary glands as a positive control for epithelial cells in which *K14^Cre^* is not active. Scale bars: 500 µm in A–C; 50 µm in D,E.
(TIF)

Figure S4 Expression of K6 (A,B) and K5 (C,D) in the dorsal tongue epithelium of adult mice. K6 expression was upregulated in the interpapillary epithelium but not in the fungiform papilla (FUP) of *K14^Cre^;Pax9^fl/fl^* mice (arrowheads in B), In contrast, K5 was normally expressed in the dorsal tongue epithelium of *K14^Cre^;Pax9^fl/fl^* mice (D). Scale bars: 50 µm.
(TIF)

Figure S5 Similar to *Prox1*, *Ascl1* is expressed in the CVP trench of wild type (arrowhead in A) but not of *Pax9*-deficient mice (B) at E18.5. Scale bars: 50 µm.
(TIF)

Figure S6 In addition to localized domains in the trenches, *Prox1* and K8 are also expressed in the apical domain of the CVP at E18.5 (arrowheads in (A) and (B)). (C) Expression of K8 in the apical domain is also seen in the *Pax9*-deficient CVP. Scale bars: 50 µm.
(TIF)

Figure S7 Histological analysis of cultured embryonic tongue explants. Beads are indicated by asterisks. (A) In the presence of Shh protein, trench formation could not be rescued in the Pax9-deficient explant and a large CVP dome developed instead. (B) An enlarged CVP did not form in mutant explants after treatment with BSA. (C) The CVP of wild type explants treated with purmorphamine (PUR) did not form an enlarged CVP dome after culture. Scale bars: 100 µm.
(TIF)

Figure S8 (A) Pax1 is expressed in taste bud cells of the adult FOP. In contrast, epithelial cells of the developing (B) and adult (C) FUP located on the dorsal tongue do not express Pax1. Scale bars: 50 µm.
(TIF)

Table S1 Measurements of CVP trenches in control and *Pax1^−/−^* embryos at E18.5. The lengths of epithelial trenches is significantly reduced in *Pax1^−/−^* embryos, whereas the width is not, the latter ruling out a general growth defect of the posterior tongue region of *Pax1^−/−^* embryos at this developmental stage.
(XLSX)
