# Supplementary figures and images for: The Formation of Endoderm-Derived Taste Sensory Organs Requires a Pax9-Dependent Expansion of Embryonic Taste Bud Progenitor Cells
Source: PLoS Genet. 2014 Oct 9;10(10):e1004709. doi: 10.1371/journal.pgen.1004709 (PMC4191947; doi:10.1371/journal.pgen.1004709)

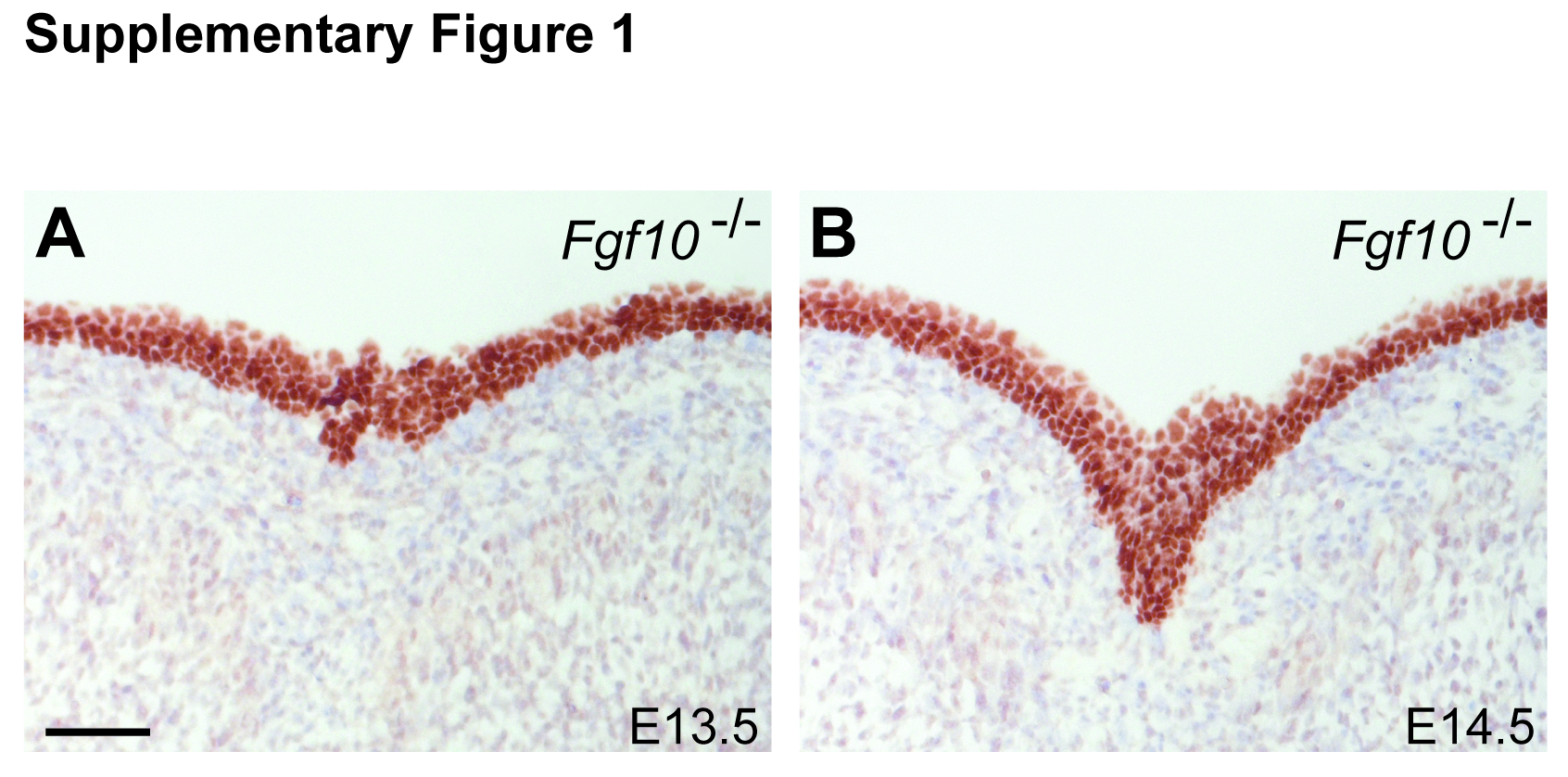

Supplement: Figure S1 — Immunohistochemical staining of Pax9 in the posterior tongue epithelium of Fgf10-deficient mouse embryos. At E13.5 (A) and E14.5 (B) Pax9 is expressed in epithelial cells of the tongue region in which the CVP normally develops. Scale bar: 50 µm. (TIF) [file pgen.1004709.s001.tif]

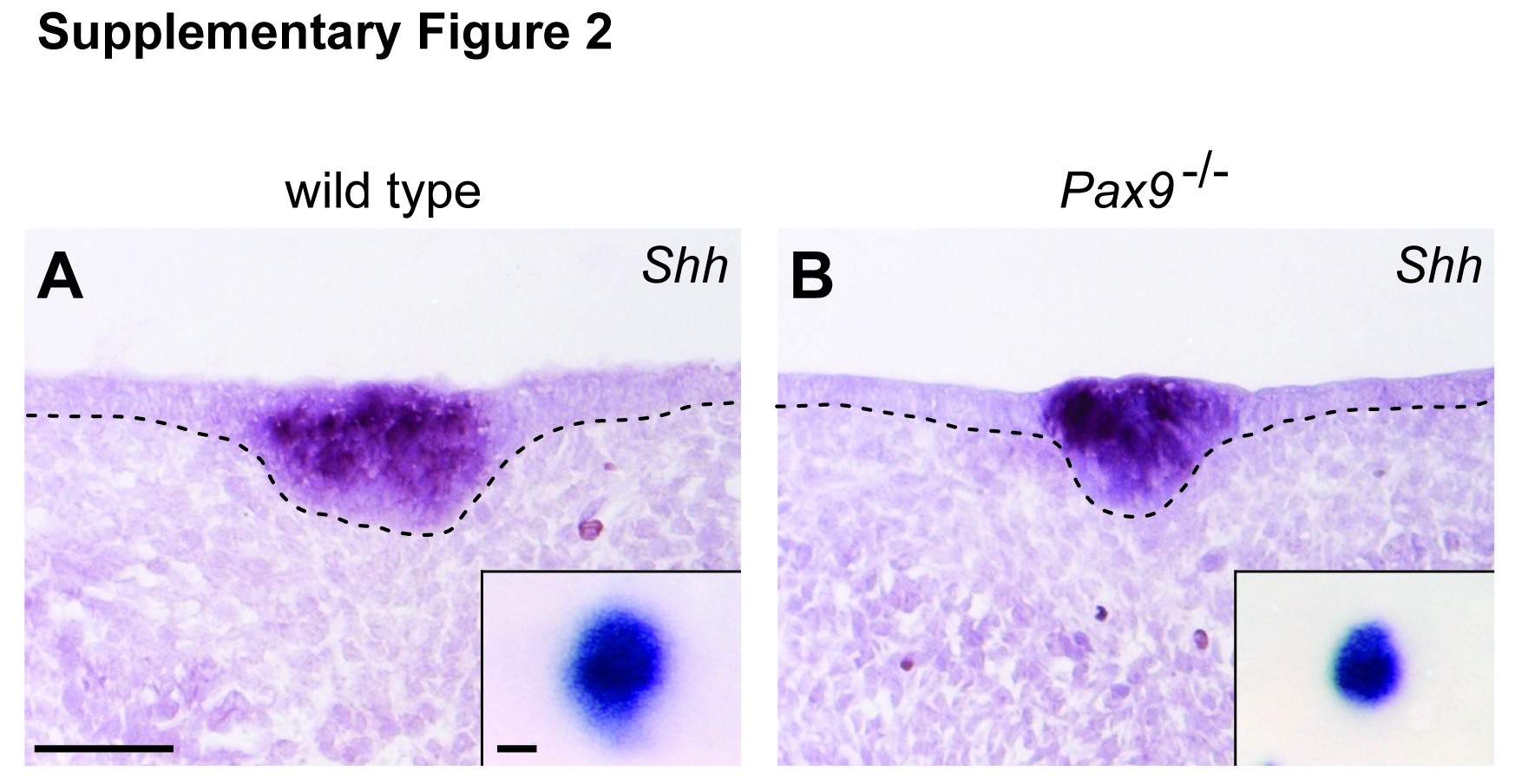

Supplement: Figure S2 — Shh expression in the CVP placode at E13.5. In situ hybridisation on sections showed that Shh is expressed in the early CVP epithelium of both wild type (A) and Pax9 mutant (B) embryos. Dotted lines outline the border between epithelium and mesenchyme and insets show Shh expression by whole mount in situ hybridisation. Scale bars: 50 µm. (TIF) [file pgen.1004709.s002.tif]

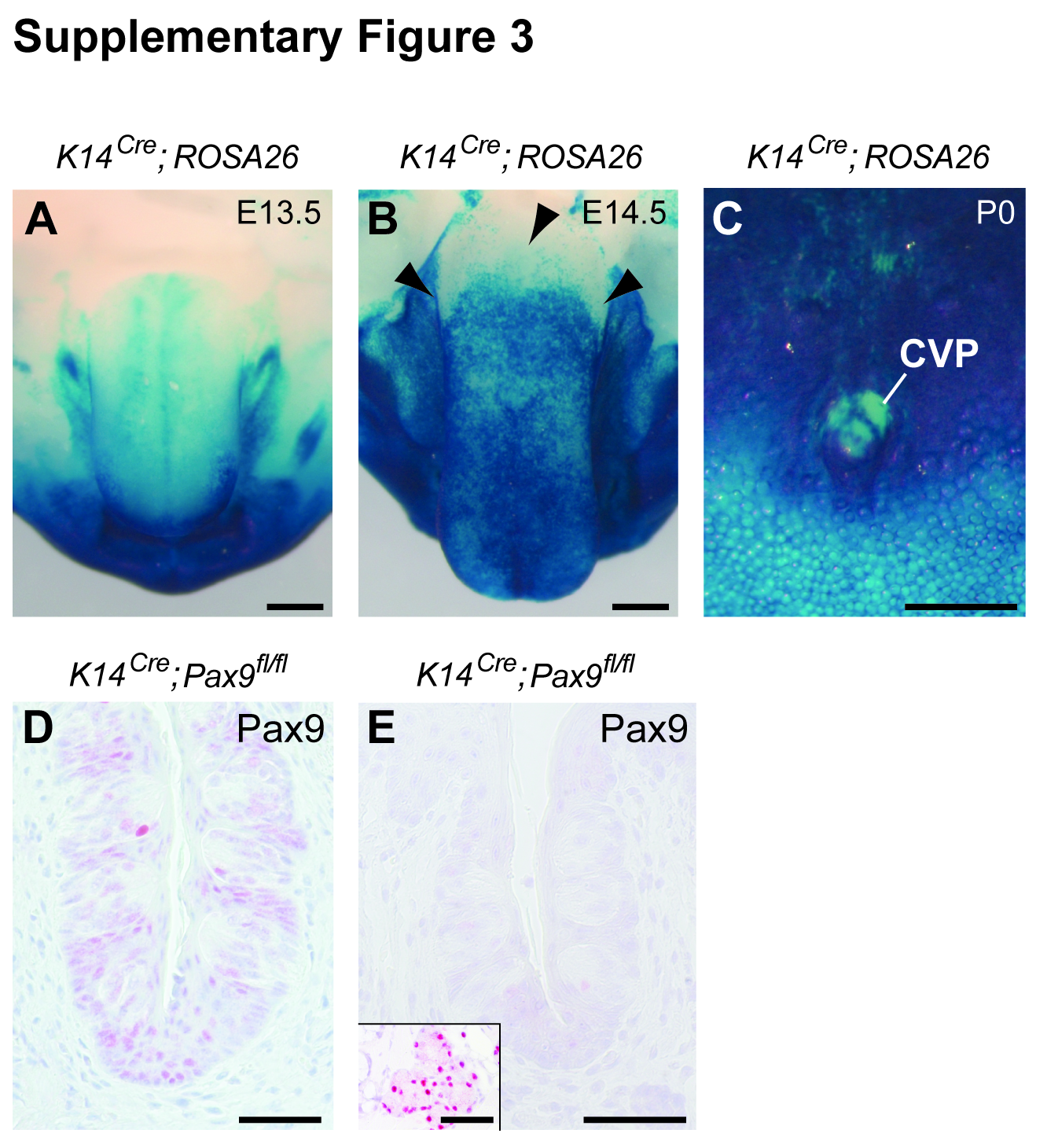

Supplement: Figure S3 — (A–C) X-Gal staining of K14Cre;ROSA26R mouse embryonic tongues at E13.5 (A), E14.5 (B), and P0 (C). Note absence of K14Cre activity in the posterior region of the tongue (arrowheads) at embryonic stages. (D,E) Pax9 immunostaining of CVP (D) and FOP (E) in adult K14Cre;Pax9fl/fl mice. Although little (CVP) or no (FOP) Pax9 protein is detectable, the morphology of the taste papillae and taste buds appears normal. Inset shows Pax9 staining in one of the minor salivary glands as a positive control for epithelial cells in which K14Cre is not active. Scale bars: 500 µm in A–C; 50 µm in D,E. (TIF) [file pgen.1004709.s003.tif]

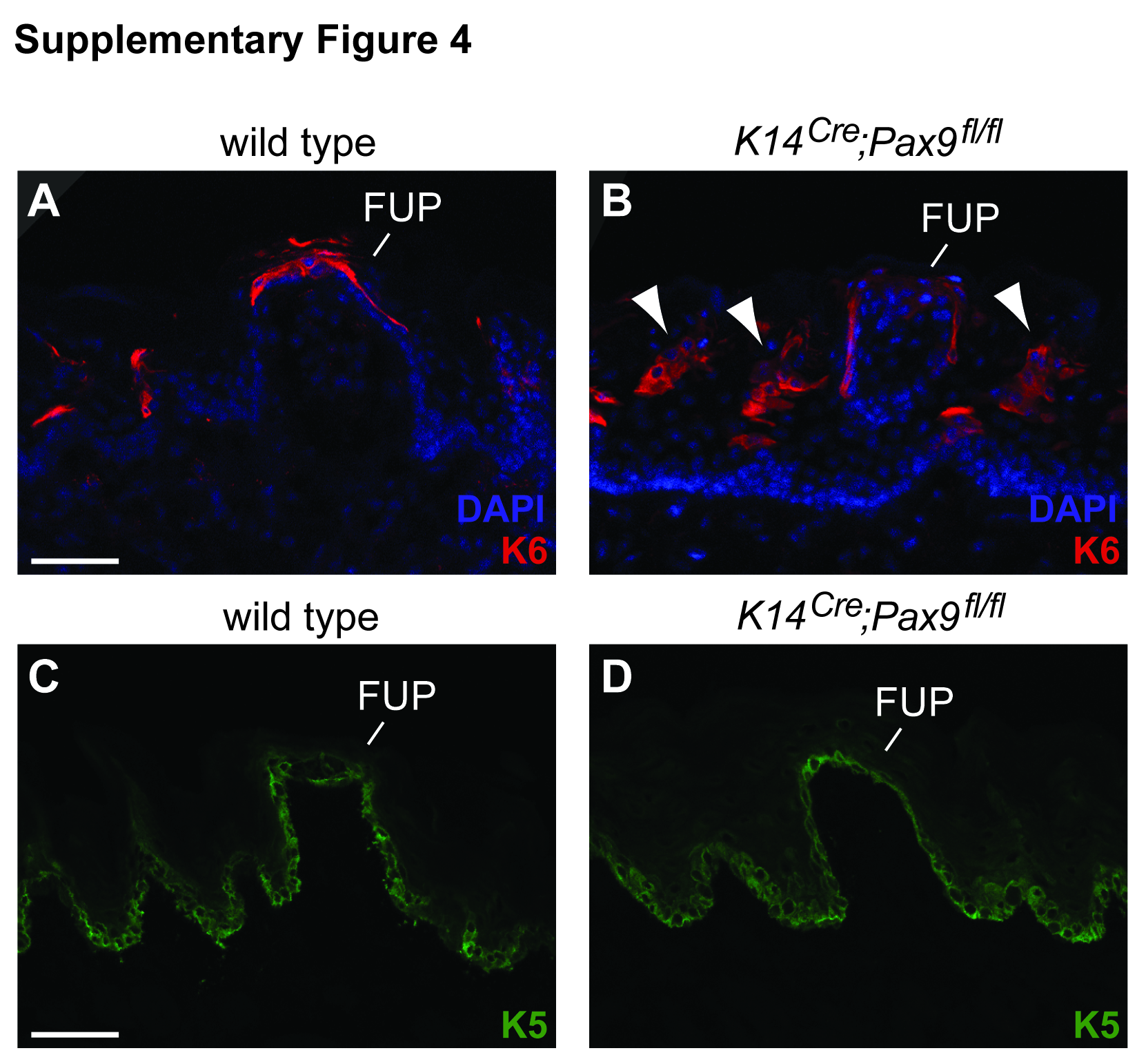

Supplement: Figure S4 — Expression of K6 (A,B) and K5 (C,D) in the dorsal tongue epithelium of adult mice. K6 expression was upregulated in the interpapillary epithelium but not in the fungiform papilla (FUP) of K14Cre;Pax9fl/fl mice (arrowheads in B), In contrast, K5 was normally expressed in the dorsal tongue epithelium of K14Cre;Pax9fl/fl mice (D). Scale bars: 50 µm. (TIF) [file pgen.1004709.s004.tif]

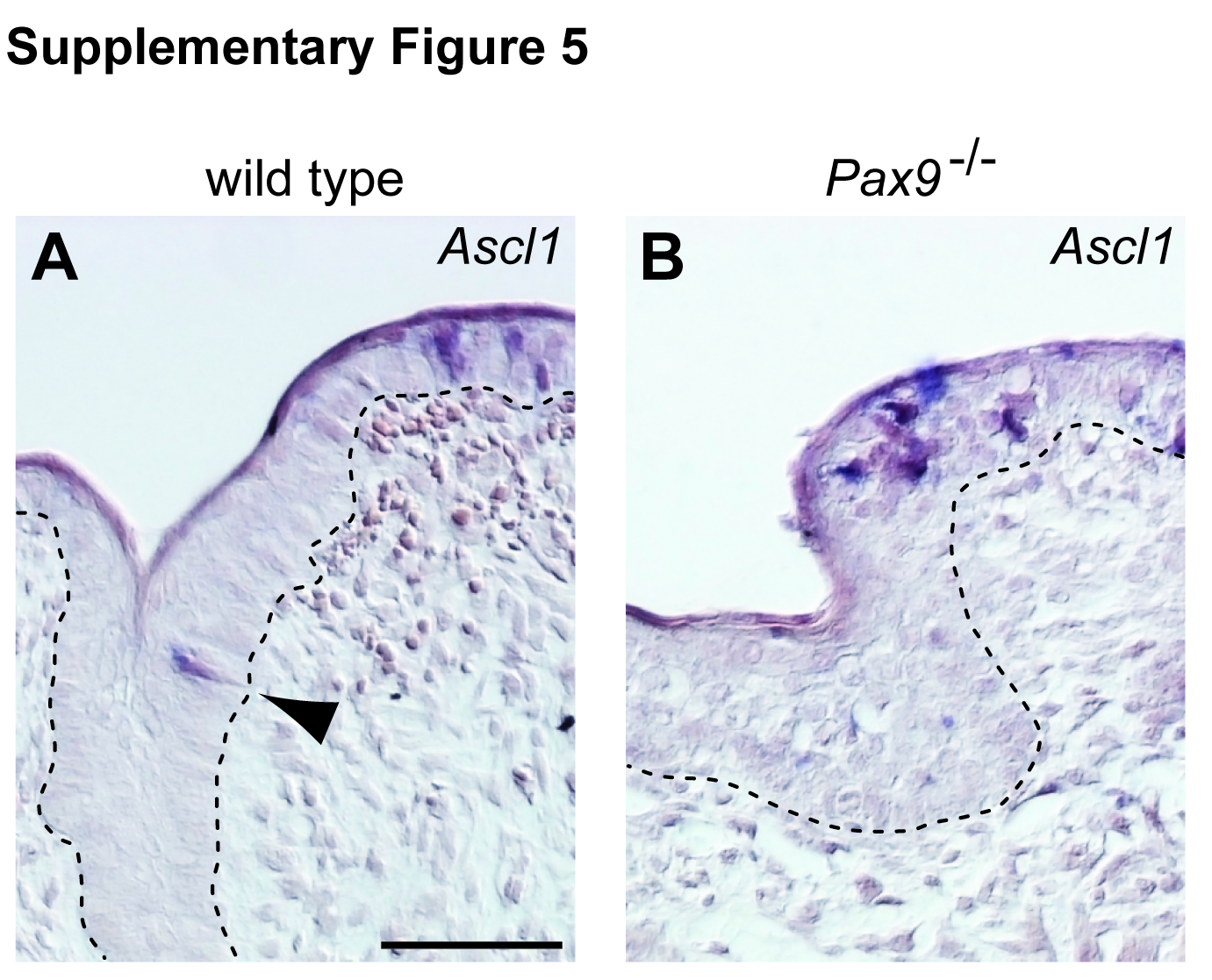

Supplement: Figure S5 — Similar to Prox1, Ascl1 is expressed in the CVP trench of wild type (arrowhead in A) but not of Pax9-deficient mice (B) at E18.5. Scale bars: 50 µm. (TIF) [file pgen.1004709.s005.tif]

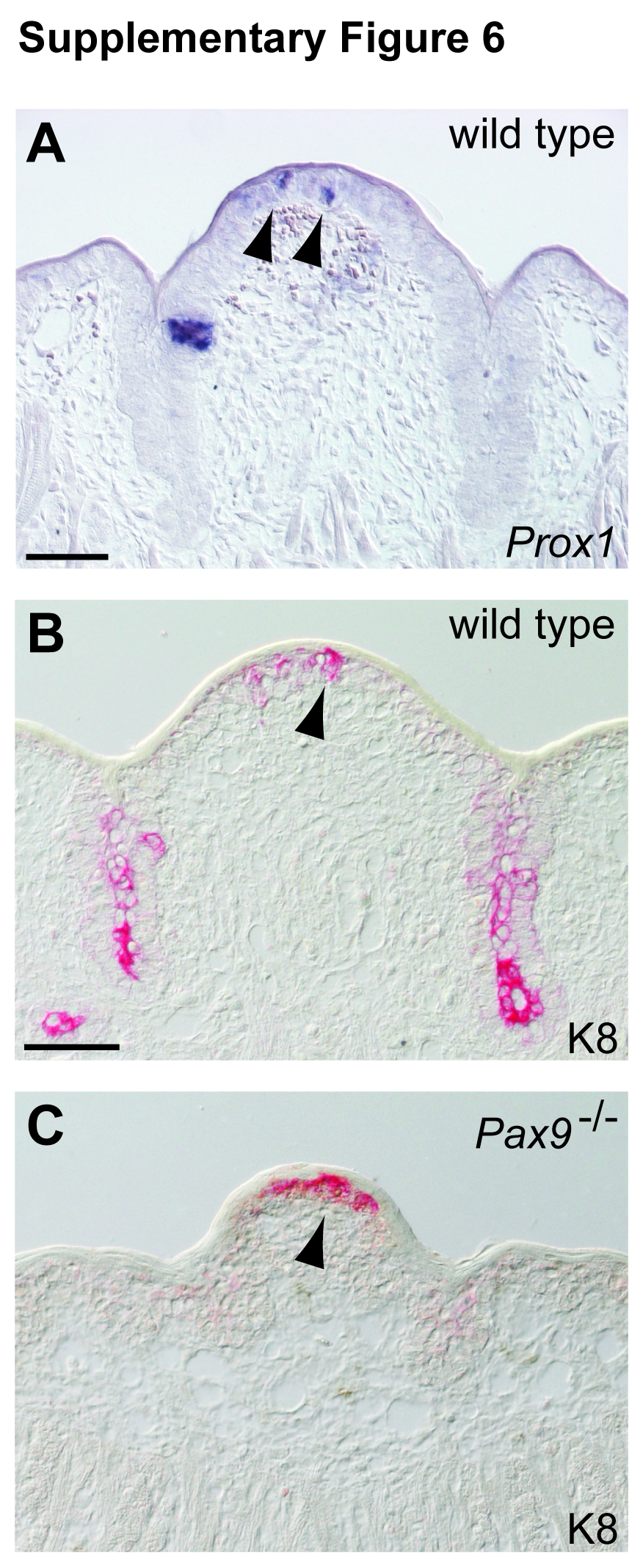

Supplement: Figure S6 — In addition to localized domains in the trenches, Prox1 and K8 are also expressed in the apical domain of the CVP at E18.5 (arrowheads in (A) and (B)). (C) Expression of K8 in the apical domain is also seen in the Pax9-deficient CVP. Scale bars: 50 µm. (TIF) [file pgen.1004709.s006.tif]

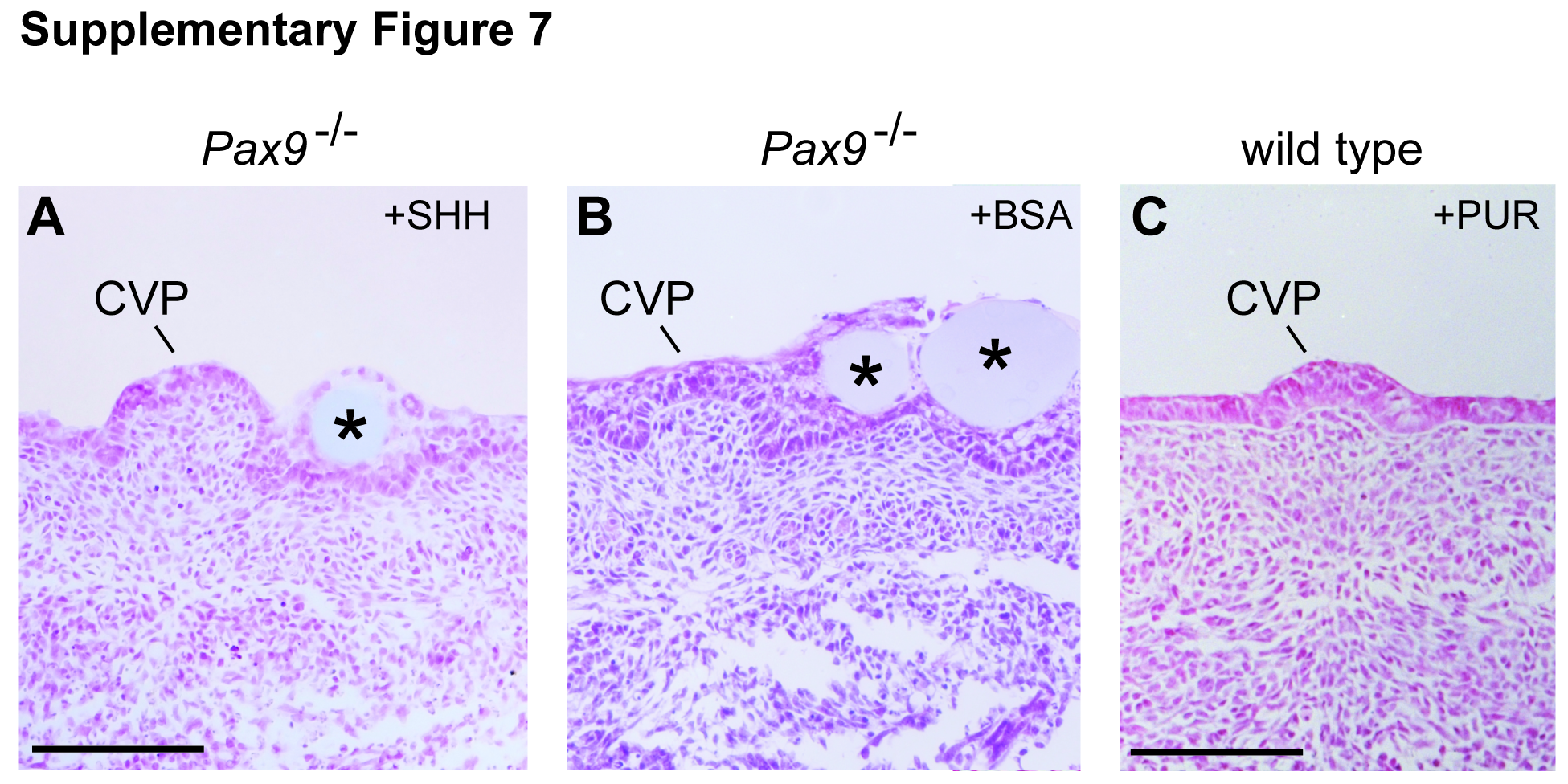

Supplement: Figure S7 — Histological analysis of cultured embryonic tongue explants. Beads are indicated by asterisks. (A) In the presence of Shh protein, trench formation could not be rescued in the Pax9-deficient explant and a large CVP dome developed instead. (B) An enlarged CVP did not form in mutant explants after treatment with BSA. (C) The CVP of wild type explants treated with purmorphamine (PUR) did not form an enlarged CVP dome after culture. Scale bars: 100 µm. (TIF) [file pgen.1004709.s007.tif]

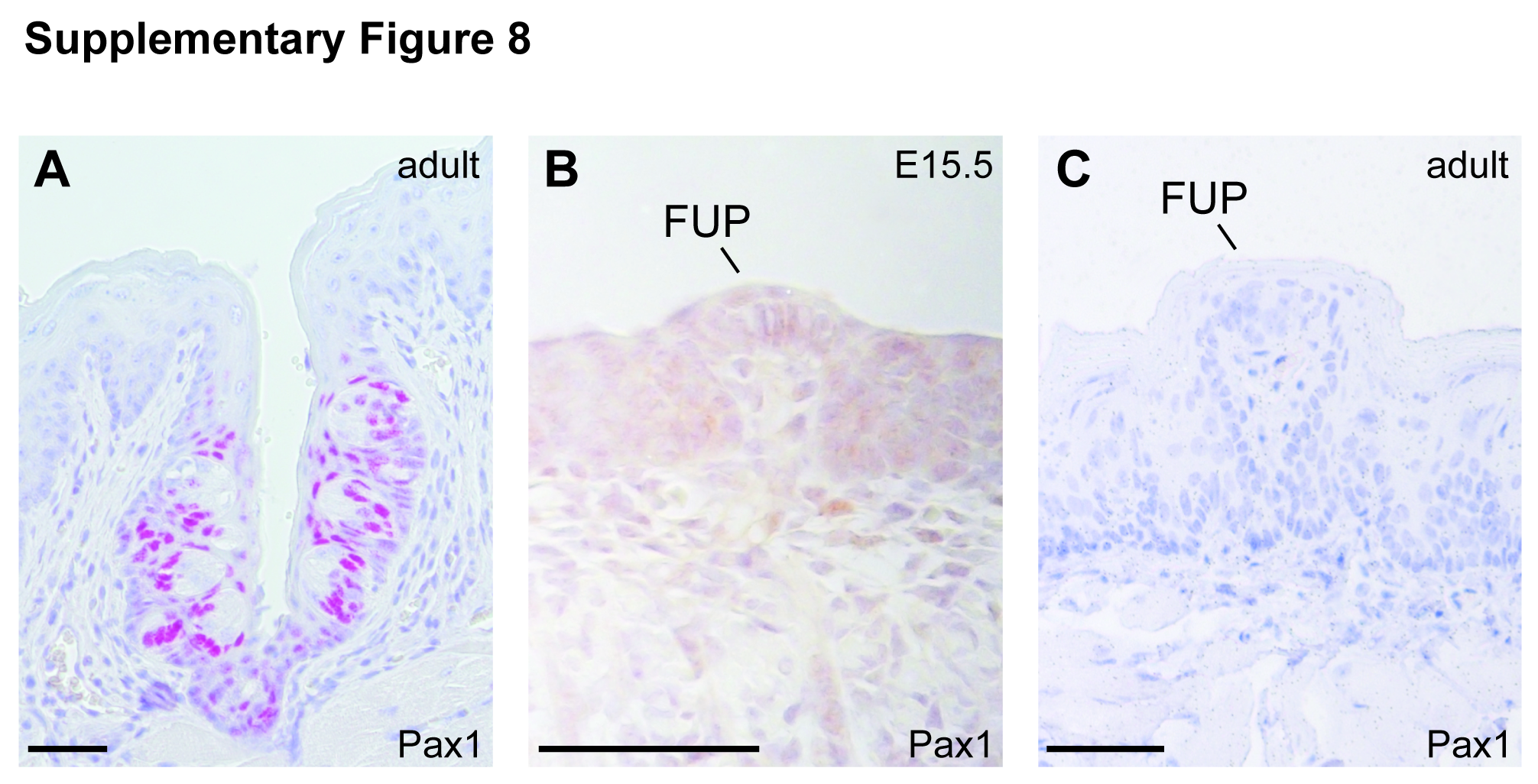

Supplement: Figure S8 — (A) Pax1 is expressed in taste bud cells of the adult FOP. In contrast, epithelial cells of the developing (B) and adult (C) FUP located on the dorsal tongue do not express Pax1. Scale bars: 50 µm. (TIF) [file pgen.1004709.s008.tif]
